# Sleep Disorders in Adults with Prader–Willi Syndrome: Review of the Literature and Clinical Recommendations Based on the Experience of the French Reference Centre

**DOI:** 10.3390/jcm11071986

**Published:** 2022-04-02

**Authors:** Pauline Dodet, Federica Sanapo, Smaranda Leu-Semenescu, Muriel Coupaye, Alice Bellicha, Isabelle Arnulf, Christine Poitou, Stefania Redolfi

**Affiliations:** 1 Centre de Référence des Narcolepsies et Hypersomnies Rares, Service des Pathologies du Sommeil (Département R3S), Hôpital la Pitié-Salpêtrière, APHP-Sorbonne, 75013 Paris, France; f.sanapo1@gmail.com (F.S.); smaranda.leu@aphp.fr (S.L.-S.); isabelle.arnulf@aphp.fr (I.A.); stefaniaredolfi@icloud.com (S.R.); 2 Institut du Cerveau (ICM), INSERM, CNRS, Hôpital la Pitié-Salpêtrière, Sorbonne University, 75013 Paris, France; 3 Rare Diseases Center of Reference ‘Prader-Willi Syndrome and Obesity with Eating Disorders’ (PRADORT), Department of Nutrition, Hôpital la Pitié-Salpêtrière, APHP-Sorbonne, 75013 Paris, France; muriel.coupaye@aphp.fr (M.C.); christine.poitou-bernert@aphp.fr (C.P.); 4 INSERM U1153, Inrae U1125, Cnam, Nutritional Epidemiology Research Team (EREN), Epidemiology and Statistics Research Center—University of Paris (CRESS), Sorbonne Paris Nord University, 93017 Bobigny, France; a.bellicha@eren.smbh.univ-paris13.fr; 5 Nutrition and Obesities: Systemic Approaches (NutriOmics), INSERM, Sorbonne University, 75013 Paris, France; 6 UMRS1158 Neurophysiologie Respiratoire Expérimentale et Clinique, INSERM, Sorbonne University, 75005 Paris, France; 7 Dipartimento di Scienze Mediche e Sanità Pubblica, Università di Cagliari, 09134 Cagliari, Italy

**Keywords:** Prader–Willi syndrome, sleep disorders, sleep-disordered breathing, narcolepsy, hypersomnia, excessive daytime sleepiness, central disorders of hypersomnolence

## Abstract

Prader–Willi syndrome (PWS) is a rare, genetic, multisymptomatic, neurodevelopmental disease commonly associated with sleep alterations, including sleep-disordered breathing and central disorders of hypersomnolence. Excessive daytime sleepiness represents the main manifestation that should be addressed by eliciting the detrimental effects on quality of life and neurocognitive function from the patients’ caregivers. Patients with PWS have impaired ventilatory control and altered pulmonary mechanics caused by hypotonia, respiratory muscle weakness, scoliosis and obesity. Consequently, respiratory abnormalities are frequent and, in most cases, severe, particularly during sleep. Adults with PWS frequently suffer from sleep apnoea syndrome, sleep hypoxemia and sleep hypoventilation. When excessive daytime sleepiness persists after adequate control of sleep-disordered breathing, a sleep study on ventilatory treatment, followed by an objective measurement of excessive daytime sleepiness, is recommended. These tests frequently identify central disorders of hypersomnolence, including narcolepsy, central hypersomnia or a borderline hypersomnolent phenotype. The use of wake-enhancing drugs (modafinil, pitolisant) is discussed in multidisciplinary expert centres for these kinds of cases to ensure the right balance between the benefits on quality of life and the risk of psychological and cardiovascular side effects.

## 1. Introduction

Prader–Willi syndrome was described for the first time by Prader, Labhart and Willi in 1956 and is considered the most common genetic cause of obesity. The incidence is estimated to be around 1 in 20,000 live births, and the population prevalence is about 1 in 50,000 [1]. The syndrome is caused by the loss of function of paternal genes on chromosome 15q11-q13 due to: (i) de novo paternal deletion of this region (65–75% of affected individuals); (ii) maternal uniparental disomy of chromosome 15 (20–30%); or (iii) an imprinting defect on the paternal chromosome resulting in the silence of the paternal alleles (1–3%) [1]. PWS is characterised by severe neonatal hypotonia and poor suck, feeding disorders (hyperphagia and lack of satiety) and early-onset childhood weight gain, often leading to obesity. Other characteristics include scoliosis, dysmorphic features, cognitive impairment and behavioural problems [1,2]. There is a large body of evidence to suggest common pathophysiology linked to hypothalamic dysfunction [2], implicated in many PWS manifestations, such as hyperphagia, multiple endocrine deficiencies (above all hypogonadism and growth hormone deficiency), temperature instability and high pain threshold. The hypothalamus is also implicated in sleep/wake regulation and in the control of breathing. Accordingly, sleep abnormalities are frequent in PWS and begin in childhood [3,4]. These disturbances include sleep-disordered breathing (SDB) and central disorders of hypersomnolence. In both cases, the principal symptom reported by the patients themselves is sleepiness, which negatively affects the quality of life [5,6,7]. The expert’s role is to classify the severity of this sleepiness according to its frequency and impact on daily activities to determine whether or not it is clinically excessive.

The aim of this review is to summarise current knowledge of the characteristics, diagnosis and treatment options of sleep disorders in PWS to provide the clinician with a practical tool for managing patients with PWS. We focus primarily on excessive daytime sleepiness (EDS), the main sleep-related symptom of PWS, detailing the clinical assessment of PWS patients. We then go on to address the two main causes of EDS in PWS—SDB and central disorders of hypersomnolence. In keeping with our background and experience, we focused on adults.

## 2. Materials and Methods

This article is the result of a long, close clinical and scientific collaboration between the National Reference Center for “Prader–Willi syndrome and rare obesities with eating disorders” (PRADORT) and the National Reference Center for Narcolepsy and Rare Hypersomnias in Pitié-Salpêtrière hospital (Sorbonne University), Paris, France. Comprehensive research of electronic literature on the PubMed/MEDLINE and Embase databases was conducted to find relevant, English-language published articles. The following medical subject headings (MeSH terms and not) were used: “sleepiness”, “excessive daytime sleepiness”, “sleep disorders”, “sleep abnormalities”, “sleep disturbances”, “sleep-related disorders”, “polysomnography”, “sleep study”, “insomnia”, “hypersomnia”, “narcolepsy”, “sleep-onset REM”, “cataplexy”, “orexin-A”, “hypocretin-1”, “modafinil”, “melatonin”, “circadian system”, “circadian rhythm”, “parasomnia”, “rhythm disorders”, “central apnea”, “obstructive apnea”, “ sleep apnea”, “sleep hypoventilation”, “sleep hypoxemia”, “hypoventilation”, “breathing sleep disordered”, “non-invasive ventilation”, “continuous positive airway pressure” [AND] “Prader-Willi syndrome”. The most relevant studies were selected by a group of expert neurologists and pulmonologists if at least one of the following topics was covered: (i) the main sleep-related symptom including sleepiness or excessive daytime sleepiness, detailing clinical examination, and questioning of patients and caregivers and the main tools for assessing sleep disorders; (ii) the SDB and its treatment, and (iii) the diagnosis and treatment of central disorders of hypersomnolence. The vastness and complexity of the subject led us to conduct a narrative review. This choice was also made on account of the great diversity of study designs in the published articles, including numerous case reports and multiple case series with a small number of patients.

## 3. Results

After eliminating 56 duplicates, 316 articles published between 1971 and 2021 were found. After reading the abstracts of each of these articles, 241 were discarded because they did not fit our inclusion criteria. We then read each of the remaining 75 articles, eliminating the irrelevant and redundant ones, to finally select only 47 articles to complete this narrative review.

### 3.1. Excessive Daytime Sleepiness

#### 3.1.1. Clinical Characteristics

Daytime sleepiness is the desire to fall asleep during the day. The expert’s role is to determine whether or not the patient is experiencing daytime sleepiness, to classify the severity of this sleepiness according to its frequency and impact on daily activities, and to determine whether or not it is clinically excessive. EDS is defined as such by the urge to sleep at abnormal times during the day, often resulting in involuntary lapses into sleep, called sleep attacks. EDS is particularly marked in passive situations (including reading, sitting or watching TV), but it can also occur in active situations (including working, eating or driving a vehicle).

EDS is the most frequent sleep-related symptom of PWS and is, at least partly, specific to PWS. In a study comparing sleep disorders in patients with different intellectual disabilities, EDS and daytime napping differentiated children with PWS from children with autism, Down Syndrome and familial intellectual disability [5]. Previous evidence from our group supports the relevance of this symptom determined through face-to-face interviews with patients and caregivers in 40 out of 60 (67%) adults with PWS [8]. Similar results on the high frequency of EDS in PWS patients are reported in a clinical review pooling 12 studies based on questionnaires or interviews [9]. EDS begins in early infancy and seems to worsen in adulthood [10,11], with significant negative consequences for the quality of life of patients and their families [4,5], on the ability to focus, and detrimental effects on daily routines and daytime activity when napping becomes too frequent. Consequently, patients with decreased activity gain weight. EDS is an invisible handicap that is often misunderstood.

In addition to EDS, the spectrum of hypersomnolence includes hypersomnia, which is defined in its strict sense as longer sleep time (more than 11 h of sleep per 24 h). However, this manifestation was reported in only 3 out of 60 patients in our above-mentioned cohort study from our research group [8].

The Epworth Sleepiness Scale (ESS) is a self-administered questionnaire routinely used to measure a patient’s sleepiness, but it has some limitations in patients with PWS, who may be too cognitively impaired to understand all the questions, even when supported by the caregivers. Indeed, in the assessment of sleepiness in adults with PWS, our group found low sensitivity of the ESS since 22% of patients determined as sleepy in the face-to-face interview had a normal (<11–24) score on the ESS [8]. On the other hand, specificity was good since only 1 patient out of 60 scored more than 10 on the ESS and did not complain of excessive sleepiness in the interview. These data suggest that ESS alone is an insufficient gauge of EDS, and the information it provides should be supplemented by face-to-face interviews with patients and caregivers.

#### 3.1.2. Sleep Recording

Overnight polysomnography combined with transcutaneous monitoring of carbon dioxide pressure is performed to assess SDB, including obstructive and central sleep apnoea and hypopnoea, as well as sleep hypoxemia and sleep hypoventilation. Night recording is followed by the multiple sleep latency test (MSLT), which corresponds to five 20-min-long nap opportunities performed from 8 am to 4 pm. An abnormal propensity to fall asleep during daytime is determined when the mean sleep onset latency is shorter than or equal to 8 min. In addition, it is considered abnormal to present two or more naps containing rapid eye movement (REM) sleep within 15 min after sleep onset (sleep-onset REM sleep periods (SOREMPs)). The combination of a daytime mean sleep onset latency below 8 min and 2 or more SOREMPs defines the narcolepsy phenotype. Abnormally long sleep time can be objectively documented using bed rest 24-h protocols, consisting of (after a habituation night followed by MSLT) second overnight polysomnography followed by full day polysomnography, allowing patients to nap as long as they can in the morning and in the afternoon [12]. A total daily sleep time greater than 11 h during 24-h bed rest condition is considered as abnormal, representing an objective marker of central hypersomnia.

EDS is, therefore, the most frequent sleep-related symptom in patients with PWS and can result from SDB, central disorders of hypersomnolence or both.

### 3.2. Sleep-Disordered Breathing: Sleep Apnoea, Sleep Hypoxemia and Sleep Hypoventilation

#### 3.2.1. Pathophysiology

The efficiency of the respiratory system in maintaining normal levels of oxygen and carbon dioxide in the blood is determined by the efficiency of ventilatory control functions and the mechanical properties of the respiratory system [13]. During sleep, ventilatory control is less effective against an increased respiratory load. Patients with PWS show impaired ventilatory control [14,15], together with multiple physical traits that contribute to suboptimal pulmonary mechanics, including hypotonia [16], respiratory muscle weakness [16], scoliosis [17] and obesity [18]. Moreover, hypotonia of upper airway muscle and fat deposition in the pharyngeal wall in association with increased upper airway collapsibility due to lung volume reduction may predispose patients to obstructive sleep apnoea. As a consequence, respiratory abnormalities are frequent and can be severe in patients with PWS, in particular during sleep [19].

- ***Ventilatory control abnormalities*.** Abnormal ventilatory control may play a significant role in the pathophysiology of SDB and has been observed in PWS patients during both wakefulness and during sleep. During wakefulness, the hypoxic ventilatory response is absent or reduced, and the hypercapnic ventilatory response is blunted in obese subjects with PWS [20]. Because arousal from sleep after rapidly developing hypoxia and hypercapnia requires intact peripheral chemoreceptor function, hypoxic [14] and hypercapnic [15] arousal responses during sleep are weakened in PWS. These elevated hypercapnic arousal thresholds during sleep predispose patients to sleep hypoxemia, sleep hypoventilation and sleep apnoea and can negatively affect the course of respiratory infections or respiratory disorders in these patients. These conditions represent a common cause of death in PWS patients [21].
- ***Hypotonia and respiratory muscle weakness*.** Hypotonia and decreased lean muscle mass characterising PWS may lead to decreased respiratory muscle strength and upper airway tone, particularly during sleep, predisposing patients to sleep hypoventilation and obstructive sleep apnoea, respectively. Moreover, the restrictive ventilatory impairment described in obese and non-obese PWS patients [16] is thought to be due, at least in part, to reduced ventilatory muscle strength.
- ***Scoliosis*.** Scoliosis, which alters chest wall mechanics, contributes to the restrictive ventilatory defect in PWS, impairing pulmonary function and increasing the likelihood of alveolar hypoventilation [17].
- ***Obesity*.** Obesity is another factor contributing to the restrictive ventilatory defect in PWS. Lung volume reduction is due to the effect of excess weight on the expansion of the chest wall and the downward movement of the diaphragm toward the abdomen. Lung volume reduction is also associated with increased upper airway collapsibility, which predisposes patients to obstructive sleep apnoea. Moreover, obesity is also associated with increased upper airway resistance in relation to fat deposition in the pharyngeal wall. Most PWS patients develop hyperphagia and morbid obesity during childhood and adulthood. Consequently, the phenotype of SDB evolves over time from predominantly central sleep apnoea in infants to obstructive sleep apnoea in older children, adolescents and adults [18]. Obesity is also considered a major cause of sleep-related hypoxemia and hypoventilation in PWS.

#### 3.2.2. Epidemiology

Adults with PWS have a higher rate of sleep apnoea, sleep hypoxemia and sleep hypoventilation compared to controls matched for age, sex distribution and body mass index (BMI) [19]. A study of 19 adult and adolescent PWS patients revealed that 21% had severe obstructive sleep apnoea (OSA, defined by more than 30 apnoea or hypopnoea episodes per hour of sleep), and 47% had sleep hypoventilation [19]. In addition, sleep hypoxemia was more common in PWS patients compared to the control group, together with stronger evidence of lower oxyhaemoglobin saturations and percentages of sleep time spent at less than 80% oxyhaemoglobin saturation [19]. In accordance with this study, in our previously mentioned cohort of 60 adults with PWS, we found 20% of patients with moderate-to-severe OSA, which was being treated in only half of the cases [8]. In addition, one patient had isolated sleep hypoxemia, whereas 8% of patients presented hypoventilation [8].

#### 3.2.3. Sleep Recording

Full-night polysomnography combined with transcutaneous monitoring of carbon dioxide pressure (PtcCO_2_) is the gold standard for the diagnosis of SDB, allowing the identification and the exact graduation of sleep apnoea, sleep hypoxemia and sleep hypoventilation, which are defined according to the following international criteria [22]: (1) untreated sleep apnoea as an apnoea–hypopnoea index > 15/h; (2) treated sleep apnoea as an apnoea–hypopnoea index < 5/h on a sleep study performed with continuous positive airway pressure (CPAP) and a CPAP adherence > 4 h/day; (3) sleep hypoxemia as >5 min of sleep with SpO_2_ < 88%; and (4) sleep hypoventilation as ≥10 min of sleep with PtcCO_2_ ≥ 55 mmHg or ≥50 mmHg in the presence of an increase >10 mmHg, as compared to the awake prone position. In the case of sleep hypoventilation, diurnal arterial blood gas analysis is used to evaluate if hypoventilation is also present during wakefulness (diurnal PaCO_2_ > 45 mmHg). At this point, an initial MSLT is recommended to objectively assess baseline EDS. In patients with behavioural disorders who cannot tolerate technically complex sleep recording with a large number of sensors, a basic assessment combining ventilatory polygraphy and diurnal arterial blood gas analysis can be proposed.

#### 3.2.4. Clinical Characteristics

Interestingly, many patients with polysomnography-confirmed OSA may not report any obvious OSA-associated symptoms [23]. In our cohort of 60 adult PWS patients, the symptoms usually related to SDB (heavy snoring, witnessed apnoea, nocturia, morning headaches) were common, although not specifically associated with the presence of objective SDB [8], showing low sensitivity in PWS. Clinicians should, therefore, anticipate and investigate OSA. Moreover, SDB in PWS patients has been associated with some behavioural disturbances, such as autism-like manifestations and impulsiveness [10].

#### 3.2.5. Growth Hormone

Failure to grow due to defects in growth hormone (GH) secretion has been described in patients with PWS [24,25]. Therefore, GH replacement therapy is currently used from childhood to improve the phenotypic appearance of subjects affected by PWS. GH treatment increases adult height, and studies in adults with PWS have consistently reported improved body composition, muscle mass, exercise capacity, as well as beneficial effects on neurocognitive functions and quality of life [23,24]. However, concerns have been raised following reports of sudden death that occurred shortly after GH initiation and were attributed to worsened OSA due to adenotonsillar hypertrophy [15]. Consequently, some randomised control trials have been conducted to investigate the effects of GH treatment on SDB. The results showed that GH does not cause a significant increase in the apnoea–hypopnoea index, indicating that GH can be safely administered [26,27]. Nevertheless, current recommendations point to the necessity of multidisciplinary evaluation, including sleep recording in PWS patients before starting GH treatment.

#### 3.2.6. Treatments

Continuous positive airway pressure (CPAP) ventilation is the first-line treatment of OSA in PWS adults, reported to be very effective in normalising respiration during sleep [28,29]. Nevertheless, compliance with CPAP treatment is low [30]. In patients with hypoventilation with or without sleep apnoea, non-invasive ventilation (NIV) is indicated. Considering the high prevalence of intellectual disability and behavioural disorders, establishing successful therapy with CPAP or, alternatively, NIV in PWS continues to pose a challenge.

#### 3.2.7. Residual Excessive Daytime Sleepiness

Treatment of OSA with CPAP leads to an improvement in EDS, according to a parental report [29,31,32,33]. Still, EDS can persist in some PWS patients even after OSA treatment [8]. There is also evidence to suggest that EDS can be disproportionate to the severity of the respiratory disturbance [30]. A retrospective study by Williams et al. found no significant correlation of sleepiness, evaluated by MSLT and ESS, with the apnoea–hypopnoea index (AHI). The authors also showed that EDS can be present in PWS patients without SDB, indicating that SDB alone cannot be the only causative trigger of EDS in PWS patients [34]. A sleep analysis by Vela Bueno et al. in nine PWS patients reported for the first time that, although most of them had symptoms of EDS, none experienced significant sleep apnoea [5].

**Figure 1:**
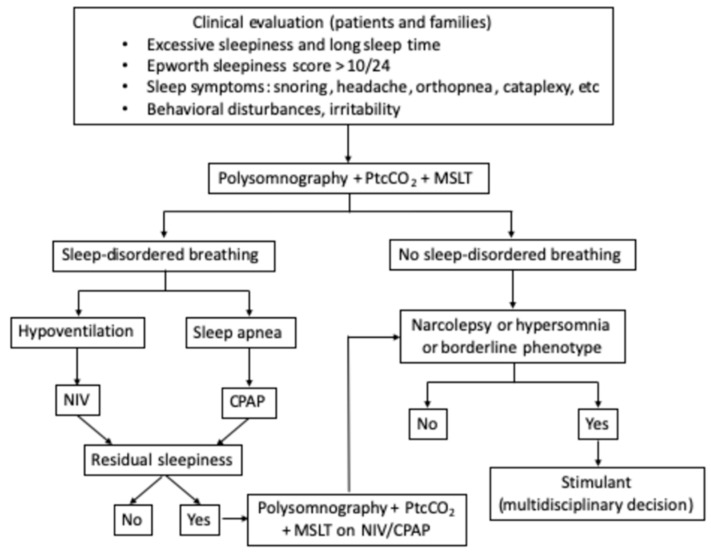
Proposed diagnostic and therapeutic algorithm for sleep disorders in adult PWS patients. PtcCO_2_—transcutaneous monitoring of carbon dioxide pressure; MSLT—multiple sleep latency test; NIV—non-invasive ventilation; CPAP—continuous positive airway pressure.

In conclusion, considering the high prevalence of SDB and its potentially severe consequences in adults with PWS, systematic screening and targeted treatment are highly recommended in these patients. Residual EDS after control of respiratory disturbance during sleep is common, and it should be properly investigated to identify and treat the other causes of EDS (Figure 1).

### 3.3. Central Disorders of Hypersomnolence

#### 3.3.1. Pathophysiology

Central disorders of hypersomnolence can result from a dysfunction of the hypothalamus, which contains two main arousal systems, the hypocretin and histamine neurons. The hypocretin neurons, involved in wakefulness and arousal [35], are damaged in narcolepsy with and without cataplexy. In PWS, many of the genes located in the lost region of paternal chromosome 15q11–q13 are expressed highly in the hypothalamus and are potential regulators of mammalian sleep and sleep-mediated metabolism [36]. This hypothesis is supported by animal models. Specifically, microdeletion of the small nuclear RNA 116 (SNORD116) cluster within the PWS locus in mice causes an EEG profile characterised by the intrusion of rapid eye movement (REM) sleep episodes during the transition between wakefulness and sleep, associated with an increase in body temperature [37]. With regard to humans, a meta-analysis confirmed that PWS patients have lower hypocretin levels in cerebrospinal fluid compared with normal subjects but higher hypocretin levels than patients with primary narcolepsy [38]. In PWS patients, decreased levels of cerebrospinal fluid hypocretin predict greater EDS [39]. A study of 8 PWS adults, 3 PWS infants, and 11 controls post-mortem failed to find hypocretin deficiency in PWS brains since no difference in the hypocretin cell number or staining intensity was observed between affected cases and controls. However, these data should be taken with caution in view of the limited sample size and lack of information about the EDS status of patients [40].

#### 3.3.2. Epidemiology and Sleep Recording

Different definitions have been used to objectively describe central disorders of hypersomnolence in PWS, which hampers a rigorous comparison of the available studies. Multiple case reports [32,41] and studies of small groups [31,42] have found excessive sleepiness in the MSLT, often associated with SOREMPs. In a review based on eight studies, Camfferman et al. reported that 29 (40%) out of 72 PWS patients (19 children and 53 adults) had a documented EDS (defined as a mean sleep onset latency < 5 min at MSLT) 9 [7]. Our previous data by Ghergan et al. in 60 adult PWS patients found an objective EDS (defined as mean sleep onset latency < 8 min at MSLT) in 13 (22%) of them [8]. This lower difference may be explained by the systematic aspect of our study. All patients underwent the sleep study even when not complaining of sleep disorder or EDS, which was not the case in the former study.

Several studies emphasised the probable deregulation of REM sleep [43,44]. In 11–21 (52%) patients with PWS [43], REM sleep abnormalities including SOREMPs, REM sleep during daytime naps, fragmented REM sleep and a decrease in the percentage of REM sleep during the night were observed.

In total, 43% (26 out of 60) of our 60 PWS adult patients in the study by Gerghan et al. displayed an isolated central disorder of hypersomnolence, which included different profiles: (i) 35% had secondary narcolepsy (MSLT ≤ 8 min and 2 or more SOREMPs); (ii) 12% had hypersomnia (including N = 2 with total sleep time > 11 h, and N = 1 with MSLT ≤ 8 min and 0 or 1 SOREMPs, *n* = 1), and 53% had a borderline phenotype (including N = 10 with ≥2 SOREMPs and MSLT > 8 min, and N = 4 with MSLT between 8 and 10 min). These established and borderline phenotypes in a genetic disease are suggestive of a hypersomnia spectrum disorder. No genetic, clinical or biological determinant was associated with the presence of central disorders of hypersomnolence [8]. The finding that the HLA DQB1*0602 genotype is strongly associated with primary narcolepsy but not with PWS [8,43,45] is of particular interest.

There are some limitations to the generalisation of our study because this kind of detailed and prolonged assessment may not be feasible in all centres, but the results here suggest that nighttime polysomnography (including CO_2_ monitoring) followed by systematic MSLT is sufficient to screen all sleep disorders in patients with PWS. Other studies need to be performed to confirm these data.

#### 3.3.3. Clinical Characteristics

EDS is the principal symptom of central disorders of hypersomnolence, but there are no specific clinical features to differentiate between the respiratory or neurological origin of EDS. However, in a central disorder of hypersomnolence, EDS tends to be more severe, with sleep lapses occurring several times per day, plus more brutal and irrepressible sleepiness, culminating in sleep attacks [8]. The dysfunction of REM sleep regulation can be associated with episodes of sleep–wake state dissociation such as cataplexies, sleep paralysis or sleep-related hallucinations. Cataplexy, which is a medical condition characterised by sudden and brief muscle weakness caused by strong emotion or laughter, has been described in some children and adults with PWS [31,43,46]. In our previous study of 60 adults with PWS, five patients exhibited partial or complete cataplexy, all showing a narcolepsy phenotype during sleep recording [8]. The other symptoms, such as sleep paralysis and hypnagogic hallucinations, are rarely reported in the literature. In our cohort, sleep paralysis was observed in two patients (one narcolepsy and one SOREMPs disorder in MSLT). None had hypnagogic hallucinations [8].

#### 3.3.4. Treatment

To our knowledge, there are no randomised studies evaluating the benefit and tolerance of a specific treatment of EDS in PWS. Consequently, the management of central disorders of hypersomnolence is only based on expert opinion and case reports. It is nevertheless necessary to take them into consideration because the expected benefits are multiple. Improved vigilance raises levels of concentration and the ability to take part in social or learning activities [47,48]. This contributes to increased self-esteem and better quality of life [47]. For this purpose, sleep experts treating PWS patients must be accustomed to using stimulants and wake-enhancing treatments in primary central disorders of hypersomnolence and must be aware of the specificity of these patients. Indeed, they are more exposed than others to the risk of adverse events due to the other symptoms of PWS, such as cognitive and behavioural disorders. In addition, all of the proposed stimulants and wake-enhancing drugs (except pitolisant) may have the potential to increase cardiovascular risk, which must be taken into consideration given the prevalence of obesity in this population. A careful assessment of the benefit/risk ratio of treatments is important. Sleep experts and PWS experts must work together closely to obtain the best benefits and avoid adverse effects. An overview of the different possible treatments is described below.

- ***Sleep hygiene.*** A regular sleep–wake rhythm with a sufficient number of sleep hours is recommended. All those factors that can degrade sleep quality should be limited (tobacco, use of electronic devices in the evening, etc.). In addition, short naps of less than an hour can be refreshing and helpful if possible. However, these sleep hygiene measures are often insufficient, and in this case, a wake-enhancing may be required.
- ***Wake-enhancing treatment.*** Modafinil and pitolisant are employed in current practice as stimulants in primary narcolepsy. These therapies have also been used for reducing EDS in PWS. To our knowledge, there are no randomised studies evaluating the benefit and tolerance of these stimulants in PWS, but there is some evidence confirming their usefulness [47,48,49,50]. A careful assessment of the benefit/risk ratio of stimulants and monitoring of the side effects are important when starting and following up treatment. In the authors’ opinion, the use of classical stimulants including methylphenidate, solriamfetol or dexamphetamine cannot be recommended at this point, in the absence of data on benefit and because they carry a higher risk of adverse cardiovascular effects than modafinil and pitolisant.

Modafinil. Modafinil is a non-amphetamine central stimulant with an unknown mechanism of action that selectively promotes alertness and is not associated with dependence or tolerance [49]. Various case reports and an open-label pilot study showed positive efficacy (reduced ESS score) without side effects in nine children and adolescents with PWS complaining of EDS (without SDB) [50]. In the mentioned study by our group, Gerghan et al. described 16 adults in the 60 PWS cohort who received a low dose of modafinil (between 100 mg and 200 mg/day). The levels of sleepiness in fourteen patients showed long-lasting improvement (ESS score decreased from 14 to 9, which was statistically and clinically significant), according to caregivers and patients. The benefit was maintained for 3.6 ± 3.1 years. The main side effects were psychological (repeated panic attacks in one patient and depressive mood in another one), occurring early after treatment start, and later (anxiety, aggressiveness, and delusional episodes), which stopped when the drug was withdrawn. Caution should be exercised regarding potential interaction with other drugs, as modafinil is an enzyme inducer. Modafinil has no impact on weight gain or loss.

Pitolisant. Pitolisant is a first-in-class histamine H3 receptor inverse agonist that enhances the activity of histaminergic neurons. This effective treatment for EDS is tolerated better than the standard-of-care drug modafinil. In three case series, although the children with PWS had not received a diagnosis of narcolepsy, parents reported that upon treatment with pitolisant, the children were not only less sleepy but also more active and more engaged [47]. Pitolisant was given to two patients who stopped modafinil because of the side effects, one reporting positive benefits and no side effects, and one lost to follow-up [8]. There is a theoretical risk of weight gain associated with increased appetite, as observed in some patients with primary narcolepsy.

## 4. Conclusions

Sleep examinations should be performed routinely on PWS patients. Indeed, as illustrated in this article, both respiratory and neurological sleep disorders are common in adults with PWS and are associated with EDS. This EDS impacts neurocognitive and psychological functions and possibly results in behavioural disorders. A clinical and instrumental assessment of sleep disorders should be carried out in a multidisciplinary expert sleep centre to customise treatment using a patient-tailored approach, with potential positive clinical and social implications for PWS patients and their families.

## Data Availability

Data sharing is not applicable.
